# Selfish centromeres and the wastefulness of human reproduction

**DOI:** 10.1371/journal.pbio.3001671

**Published:** 2022-07-05

**Authors:** Laurence D. Hurst

**Affiliations:** 1 Wissenshaftskolleg zu Berlin, Berlin, Germany; 2 The Milner Centre for Evolution, University of Bath, Bath, Somerset, United Kingdom

## Abstract

Many human embryos die in utero owing to an excess or deficit of chromosomes, a phenomenon known as aneuploidy; this is largely a consequence of nondisjunction during maternal meiosis I. Asymmetries of this division render it vulnerable to selfish centromeres that promote their own transmission, these being thought to somehow underpin aneuploidy. In this essay, I suggest that these vulnerabilities provide only half the solution to the enigma. In mammals, as in utero and postnatal provisioning is continuous, the costs of early death are mitigated. With such reproductive compensation, selection can favour a centromere *because* it induces lethal aneuploidy: if, when taken towards the polar body, it instead kills the embryo via aneuploidy, it gains. The model is consistent with the observation that reduced dosage of a murine drive suppressor induces aneuploidy and with the fact that high aneuploidy rates in vertebrates are seen exclusively in mammals. I propose further tests of this idea. The wastefulness of human reproduction may be a price we pay for nurturing our offspring.

## Abstract 2

Why do so many human embryos have the wrong number of chromosomes? So-called ’selfish centromeres’ and the fact that new embryos can be produced may provide an answer.

## The evolutionary enigma of human aneuploidy

Human reproduction is “extraordinarily wasteful” [1], largely owing to aneuploidy. Possibly more than 70% of oocytes are aneuploids [2,3] and about 30% to 60% of preimplantation human embryos are [4] (see Box 1: Glossary), the great majority being spontaneously aborted without a recognized pregnancy [4]. In cases where the pregnancy is clinically recognised, around 10% to 20% result in early spontaneous abortion [1] of which more than 35% are owing to aneuploidy [4]. Owing to this high in utero mortality, only 0.4% of human pregnancies result in an autosomal trisomy at term (all monosomies fail), these presenting as Down syndrome (chr 21), Edwards syndrome (chr 18), and Patau syndrome (chr 13) [5]. Nearly all cases of recognised pregnancies of these latter 3 also die in utero (49% to 87%) or shortly after birth, prior to weaning [6,7], trisomy 21 being an exception with 84% to 92% 1 year survival with advanced healthcare [7]. Survival rates in sub-Saharan Africa are much lower, largely owing to congenital heart defects [8]. Aneuploidy is thus both remarkably common and almost always associated with preweaning mortality. How can we, evolutionarily speaking, account for such enigmatically high rates of aneuploidy and associated early mortality?

**Box 1: Glossary**

**Aneuploid:** having an abnormal number of particular chromosomes (e.g., for humans 45 or 47 chromosomes as opposed to the normal 46). Distinct from polyploidy which is a change to the number of full sets of chromosomes.

**Centromeric drive:** non-mendelian segregation owing to an allelic form of the centromere with the ability to be transmitted to more than 50% of embryos in a heterozygote with alternative alleles.

**Cytoplasmic male killer:** maternally transmitted cytoplasmic factor (commonly an intracell bacterium) that kills male offspring.

**Euploid:** having the canonical number of chromosomes for the species concerned (*N* = 46 for humans).

**Kinetochore:** a disk-shaped protein structure assembled at the centromere to attach microtubule polymers from the spindle.

**Meiosis:** the process by which a diploid cell divides to produce haploid progeny cells (e.g., sperm or eggs).

**Monosomic:** aneuploid individual missing 1 copy of 1 particular chromosome. Not to be confused with haploidy, 1 complete set of chromosomes.

**Nondisjunction:** the process of incorrect chromosomal assortment through meiosis leading to aneuploid gametes (i.e., gain or loss of a given chromosome).

**Polar body:** a small product of a meiotic segregation event that has no reproductive future.

**Prisoners’ dilemma:** a “toy” game theoretical model employed to understand conditions for the evolution of cooperation. Two prisoners, who cannot communicate with each other, are given the option of cooperating with each other (not saying who committed a crime), in which case they both get reward *R*, both betraying the other, in which case they both get punishment payoff *P*, or one betraying one not. The co-operator then gets *S* (sucker’s payoff), while the defector gets the temptation payoff, *T*. The game specification requires *T>R>P>S*.

**Reproductive compensation:** any process in which death of offspring is associated with parental savings (in time or resources), enabling reinvestment into viable offspring.

**Selfish genetic element:** any allele with exclusively vertical transmission that can deterministically increase in frequency in a population while also being deleterious. Also referred to as genomic parasites, genomic renegades, selfish elements, etc.

**Trisomic:** aneuploid individual with 3 copies of 1 particular chromosome. Not to be confused with triploidy, 3 complete chromosome sets.

## Maternal meiosis I and centromeres are implicated

A possible clue comes from biases in the origin of the nondisjunction. While in sperm, the aneuploidy rate is only around 1% to 4% [4], in maternal meiosis the rate is 30% to 70% [4], these mostly occurring in meiosis I [9–11]. The high numbers of early embryonic meiosis I errors contrasts with what is seen in, for example, yeast (*Saccharomyces cerevisiae*) in which meiosis I aneuploidy is vanishingly rare [12].

In addition, multiple lines of evidence implicate centromeres in meiosis I aneuploidy: Centromeric cohesion [13] predicts maternal age aneuploidy effects, meiosis 1 kinetochore instability, causing centromeric spindle assembly problems, leads directly to aneuploidy [14] and dosage of centromeric binding proteins mediate aneuploidy induction in mice [15].

The structure of maternal meiosis I (MMI) is such that a mutant centromere can easily come about that increases its transmission rate in heterozygotes [16–20] and so readily spread through a population. It is then suggested that such selfish mutants could somehow be associated with aneuploidy [16], although the coupling is typically vague [21,22]. Here, I suggest that MMI vulnerability may be only half of the solution: Owing to the structure of MMI, a mutant centromere can both easily appear that results in aneuploidy and, in a species with reproductive compensation, can readily invade *because* it causes lethal aneuploidy (S1 File). We may indeed be especially prone given our long parental care. Here, I lay out the argument, review what evidence is available, but more importantly suggest possible follow-on studies to test the idea. I start by laying out the nature of these 2 vulnerabilities, reproductive compensation and MMI anatomy.

## What reproductive compensation is and why mammals are especially affected

In a mammal with multiple embryos in a brood, if one dies, the surviving progeny have less competition for resources and so can become fitter [23–25]. In this sense, the death of the embryo is partially compensated through the increased fitness of survivors, such that decreased brood size is associated with larger mean progeny size [23–25]. Investment to subsequent broods or time to next reproductive effort can also be affected [26,27]. When there is only 1 conceptus per pregnancy (e.g., commonly in humans) and this dies, there is both a saving of resources which can be granted to the next progeny and a much shorter time to the next reproductive effort [28]. If death or sterility is to occur, selection can favour the earliest possible in utero mortality to maximise this compensation [29,30].

Human twinning data allows a broad estimation of the magnitude of some of the intrabrood effects. Human singletons weigh about 40% more than twins (3,296 g versus 2,336 g [31]). Thus, assuming all else is equal, a twin pregnancy in which 1 embryo dies early realises about 70% of the investment that there would have been, not the 50% expected with no compensation. From weight-by-survival curves [32], this 40% increase in weight of the singleton equates to about a 10% increase in relative fitness of the singleton (S1 File). In both fitness and resource terms, the death of the twin is partially compensated.

Importantly, this mammalian provisioning is continuous, meaning that if an embryo dies, the investment is curtailed and can be redirected. Of all species, mammals, with in utero care and postnatal weaning, thus have the greatest potential for reproductive compensation [29,30,33,34]. The polar opposite to mammals are those fish in which eggs are externally fertilised and immediately released into a large body of water (i.e., no parental care, no resource competition between the young fish). Embryo death here has no effect on any present or future offspring. Birds are more like fish than mammals in that the mother provides all the costly [35] resources for the egg (yolk, etc.) up front. Birds will also incubate inviable eggs whether as part of a larger clutch [36] or singly [37], and incubation is a major cost of reproduction [38]. Intra-egg mortality thus does not necessarily save costs associated with time (sitting on the egg) nor resources (intra-egg embryo provisioning). Embryonic mortality will, however, have some degree of compensation in birds as post-hatch parental care effort can be reduced or there could be benefit from reduced sib-competition. Indeed, in some species some chicks can only survive if another dies [39].

I suggest that mammalian compensation leaves us vulnerable to selfish centromeres that induce aneuploidy, because they enable this redirection of resources, the selfish centromere being a recipient.

## The structure of maternal meiosis I is vulnerable to selfish centromeres

**Fig 1:**
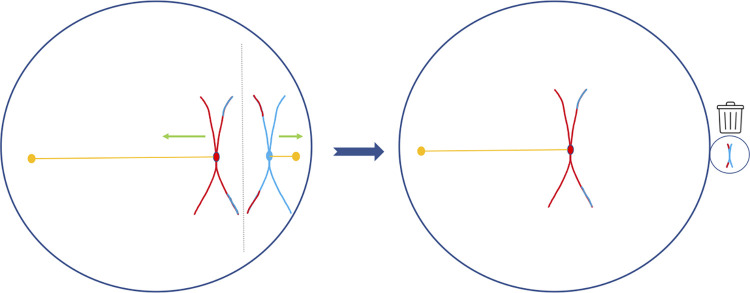
The 3 asymmetries of maternal meiosis I. Here, we consider only 1 of our 23 pairs of chromosomes. Each chromosome, as inherited from the mother or father, is coloured either red or blue. The first step of meiosis I is to replicate each chromosome, generating an X-shaped structure pinched at the centromere (circle at the pinch point of the chromosomes). After meiosis I crossing-over, the chromosome arms swap so causing a change from blue to red of vice versa. The chromosomes in the left figure are seen at this stage. However, with no crossing over at the centromere, segregation during meiosis I always segregates unrelated maternal and paternal centromeres (the relatedness asymmetry). The centromeres attach to spindle microtubules (orange line) that pull the relevant chromosome in 1 of 2 directions. Here, the centromeres and associated kinetochores are shown as 1 entity per chromosome. In reality, there are 2 that tend to co-orient on the same chromosome. One centromere will be dragged to the small polar body, this having no reproductive future (illustrated by a bin), the other to the egg pole, this one then entering meiosis II and having a future. This is the fate asymmetry. The small size of the polar body compared to the egg creates a size asymmetry. The chromosomes align across the meiotic plate (dotted vertical line). Owing to the size asymmetry, this is often located towards where the polar body will appear. This creates a vulnerability in that centromeres can in principle gain information as to which pole they are being dragged, owing to gradients that run across the egg.

The other vulnerability comes from the structure of MMI. MMI is unusual because it is asymmetric in 3 regards: fate, relatedness, and size (Fig 1). Only one of the 2 products of the first division has a future (the egg), the other (the polar body), being a transmission dead end. The same is not true in male meiosis where all products of meiosis (sperm) are viable. This fate asymmetry sets the stage for competition between unrelated homologous chromosomes to be incorporated into the egg. Importantly then, the first division segregates the unrelated maternally and paternally derived centromeres. This lack of relatedness holds because centromeres have no crossing over (and assumes a history of outbreeding). Evolutionary competition between centromeres is thus expected in MMI segregation but not MMII segregation. The size asymmetry (a small polar body and a large egg) requires that the spindle move closer to the periphery of the egg when division occurs. There is thus the potential for a chromosome to have information as to which pole it is being pulled towards [40–42]. This can enable exploitation of the prior 2 asymmetries [40–42].

Evolutionary arguments for the trisomy/aneuploidy enigma [16,21,22] have centred (implicitly [21,22] or explicitly [16]) on the notion that, owing to these asymmetries, MMI (of all species, not just mammals) is especially vulnerable to centromeric drive [16–20] and that, somehow, such drive is related to aneuploidy. Centromeric drive is a process in which a centromere is more commonly incorporated into the egg rather than the polar body than its unrelated competitor centromere [16–20]. Centromeres are hotspots for such drive as they both have guaranteed lack of relatedness in meiosis I and the opportunity. Centromeric drive can, for example, be a consequence of expansion of the centromeric satellite array, which thus gains the ability to attract more CenH3 nucleosomes than its counterpart on the homologous chromosome. More such nucleosomes result in a “stronger” kinetochore, which preferentially captures egg pole microtubules [41,43]. Some centromeric drive systems employ conditional behaviour: If a centromere is being dragged to the polar body, this centromere can “flip,” i.e., detach itself from the polar body oriented microtubules and try to become oriented to be taken to the egg [41]. Flipping events are strongly biased (81%) to detach larger centromeres from the cortical (polar body) side and reorient then towards the egg side [41]. Such behaviour requires some “information” source by which chromosomes might “know” which pole they are heading towards. Signalling from the oocyte cortex leads to asymmetry in a posttranslational modification of tubulin, tyrosination, causing an asymmetry between poles [40–42].

Centromeric drive causes a deviation from Mendelian ratios in the progeny. At the limit, when a centromere (*D*) drives against the wild type (*d*) in *Dd* females, all of the haploid eggs are *D* when there would otherwise be 50:50 ratios. Owing to this distortion, even a deleterious driving centromere can, when rare, readily increase in frequency in a population [18,20,39,44] (see also S1 File). Such rapid invasion will reduce variation around centromeres. In humans, there is evidence for this [45,46] and, given the absence of genes in the vicinity, selfish centromeric drive is considered a possible explanation [45,46].

Centromeric drivers are assumed to be costly, and hence, one class of selfish genetic element [47]. If so, we might expect bouts of invasion followed by suppression to reduce costs. For example, just as attraction of Cen3H can enable drive [41,43], so too any harmful effects are countered by the adaptive evolution of CenH3 [20,48]. The centromeric interacting Bub1 is a drive suppressor in a mouse hybrid model [41]. Consistent with the expected antagonistic coevolution, drive and suppressing elements are fast evolving [17–19,41,49–51].

## Coupling centromeric drive and aneuploidy

Axelrod and Hamilton [21] proposed the first of three [16,21,22] evolutionary models coupling drive to aneuploidy. They approached the more general problem of when, in the Prisoner’s Dilemma game, two individuals should cooperate. The game allows the two individuals (centromeres in the present case) to either cooperate or defect (Box 1). They found that persistence of cooperation requires a potential for future interactions (iteration) [21]. “Fair” (mendelian) segregation they considered a consequence of cooperation between chromosomes. As menopause approaches, hence an end to iteration, the trend to defect (drive) would, they suggested, increase. The rationale for a coupling between drive and trisomy was, however, vague, it being argued that “an extra chromosome in the offspring could be the occasional result” [21]. If so, their model could potentially explain why Down syndrome, and trisomies more generally, are more common when mothers are older [21].

The logic of this model was questioned by Day and Taylor [22] who noted that, as human menopause forces a fixed end to the iterations, the stable solution is to always defect/drive [22]. Their modified model [22] also presumes that trisomy is some incidental by-product and that stronger drive entails increased trisomy. If stronger drive is also associated with increased female mortality, then increased trisomy rates with age are predicted [22].

Zwick and colleagues [16] criticised this model for its failure to explain both chromosome loss events and heritable variation on the nondisjunction rate. The model might also be criticised for its ad hoc assumption that stronger drive results in lower female viability. Indeed, the criticisms are coupled in that to derive a polymorphism-free equilibrium, the model needs to make this ad hoc assumption. Instead, Zwick and colleagues [16] suggest a model in which “female-specific nondisjunction acts as a deleterious effect countering meiotic drive.” They also note the possibility for multiple drivers and suppressors leading to complex dynamics [16]. Such complex dynamics are consistent with fast evolution of centromeres and their suppressing elements [17–19,41,49–51].

## If there is reproductive compensation, aneuploidy can be beneficial to a selfish centromere

The above models suggest either that aneuploidy is somehow an incidental by-product of drive or possibly suppressive [16,21,22]. It is the nature of these models that females of all species with asymmetric meiosis should be vulnerable to centromeric drive and hence that all such females should be vulnerable to aneuploidy. Here, by contrast, I suggest that if there is reproductive compensation, it can be in the best “interests” of a selfish centromere to induce aneuploidy. Aneuploidy should then be particularly associated with asymmetric female meiosis in species with reproductive compensation.

Consider the consequence for selfish centromeres of having information as to which pole it is heading (cf. conditional flipping [41]). We assume the wild-type centromere is not distorting in any manner. Now put yourself in the place of a rare (in the population) aneuploid-inducing centromere. This will initially be found in a heterozygote. Half the time, the new centromere will, by chance, be taken to the egg pole. It wins. The other half of the time, the centromere is taken towards the polar body (Fig 1). Once the selfish centromere “knows” this, it has nothing to lose: No matter what it does, it cannot be worse off than being destroyed in the polar body. If the centromere now detaches from the spindle [41] and reattaches to the egg pole, it could create a triploid. As it wasn’t going to be in the viable egg anyway, it has lost nothing. More importantly, if the aneuploidy-induced destruction of the embryo has associated reproductive compensation, the selfish centromere again evolutionarily wins (S1 File). In humans, the mother may, for example, reproduce again immediately. The centromere then has a 50:50 chance of being transmitted in the next reproductive event (as opposed to the zero chance if it does nothing). Alternatively, as in mice, resources may be redistributed to surviving brood sibs [23] that will disproportionately be bearers of the selfish centromere (S1 File). Note that it isn’t the very centromere that kills the embryo that gains—it is in a dead embryo/oocyte—but rather its clonal identical relatives in subsequent reproductive efforts or the same brood, this being intraclonal kin selection [52].

With estimated levels of mammalian reproductive compensation (e.g., 10% fitness increment), invasion conditions for a conditional aneuploidy-inducing centromere are broad (S1 File). The compensation associated with aneuploidy could also be part of the selective advantage of the “flip” strategy. By flipping a selfish centromere could cause simple segregation distortion (it goes to the egg pole and the other centromere to the polar body) or both could end up in the egg. If there is reproductive compensation, it wins either way (S1 File).

One criticism of some prior drive-aneuploidy models is that they were inconsistent with chromosome loss events [16]. For the current suggestion, in terms of the fitness consequences, it is irrelevant as to whether embryo mortality is via chromosome gain or loss. Put differently, a selfish centromere would benefit from a strategy of “if I’m going to the polar body, I’ll poison the embryo by taking the other chromosome with me” (i.e., monosomy) and from “if I am going to the polar body, I’ll stay in the embryo and poison it” (i.e., trisomy). Similarly, a driving centromere that sometimes is also associated with chromosome loss or gain will selectively gain from embryonic mortality in both instances, so long as the species has reproductive compensation. The model is thus not inconsistent with chromosome loss events.

**Fig 2:**
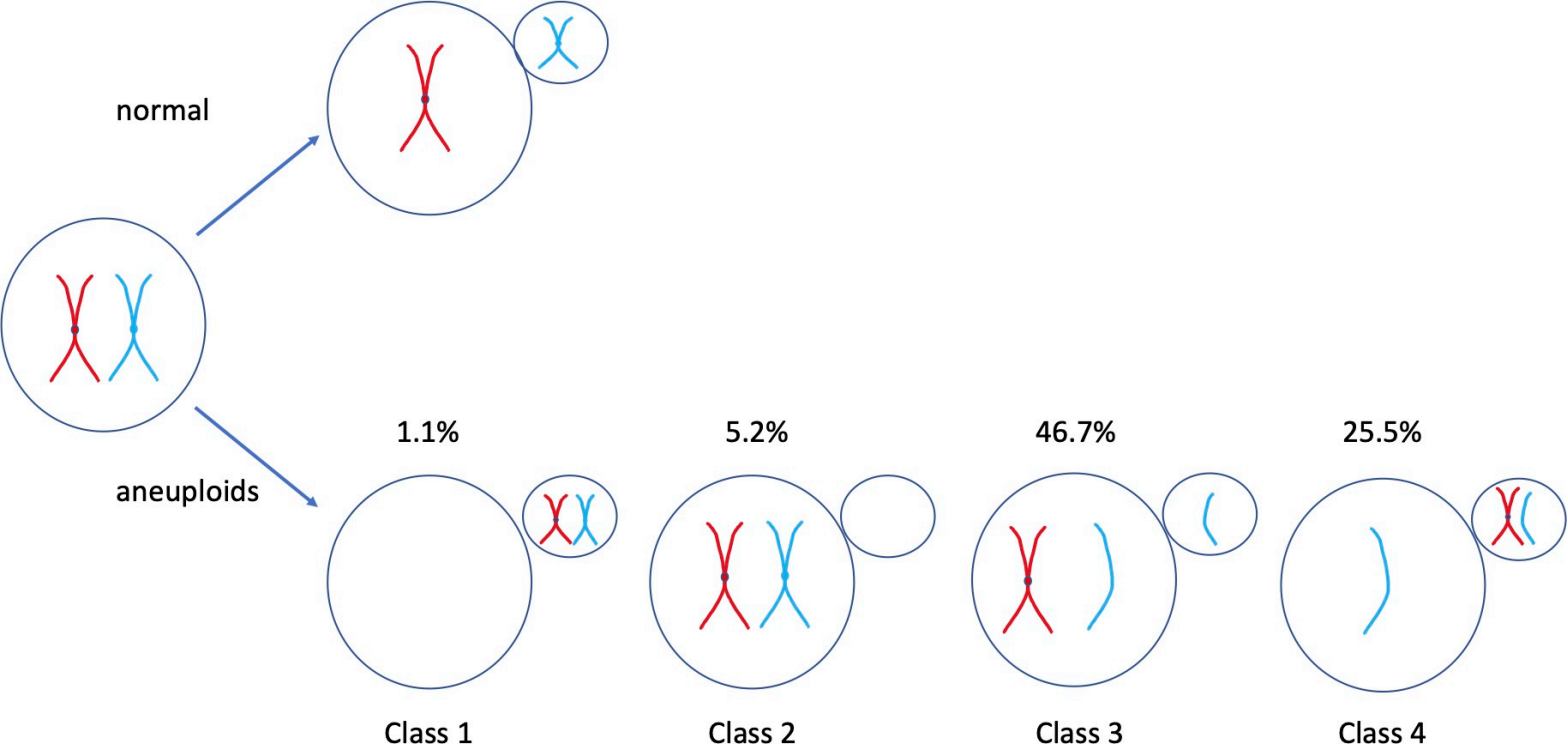
Relative rates of meiosis I error in 20,000 human oocytes (data from [53]). The percentages shown are the percentages of all errors that are each type of error. Missing is class 5, complex errors (21.5%).

Prediction of the expected ratio of chromosome loss to chromosome gain events (in oocytes) is then largely down to mechanistic concerns. Mechanistically, human aneuploidy is owing to abnormal kinetochore–microtubule interactions [14]. Human kinetochore pairs (1 pair per centromere), although typically co-orienting if on the same chromosome, routinely attach to both poles in early meiosis I [14]. When the attachments go unresolved by anaphase 1 (merotelic attachment), aneuploidy is the result [14]. Mechanistic details are different in mice where spindle instability is rare [14]. There are 5 classes of aneuploidy error in meiosis I [53] (Fig 2). Analysis of some of the shorter chromosomes (13, 16, 18, 21, and 22) in 20,000 human oocytes indicates that both sets of chromosomes going to the polar body (0:4 segregation, nullisomy) is rare (1.1% of errors) [53] (Fig 2, class 1) with further studies finding no examples [54]. By contrast, both chromosomes pairs (4:0, class 2, 5.2%) or a full chromosome and a chromatid (3:1, class 3, 46.7%) being retained in the oocyte after meiosis I are relatively common [53]. Retention of 1 chromatid (1:3, class 4, 25.5%) in the oocyte or complex events (class 5, 21.5%) account for the remainder of the errors. In analysis of larger chromosomes, the ratio of 3:1 to 4:0 events is strongly skewed to 4:0 events [54], but in all cases, the symmetrical events (3:1 verses 1:3, 4:0 versus 0:4) are skewed to oocyte incorporation (i.e., 4:0 >> 0:4, 3:1 > 1:3).

The current model may, I speculate, provide an additional (beyond purely mechanistic issues) rationale for this bias to oocyte incorporation of symmetrical events. From an evolutionary point of view, the process is not symmetric: There is a greater “incentive” for the kinetochores that have preferentially attached to the polar body microtubules to detach from these. Such conditional uncoupling from the polar body microtubules [41] is likely to lead to these kinetochores being attached to the egg pole [41] and to create a temporary bias to egg pole attachments (4:0). These could easily be resolved as 4:0 or 3:1, if anaphase I resolution hasn’t occurred in time, or 2:2 (either a flip or failed flip). A 1:3 event will then require 3 of 4 kinetochores to switch or incomplete microtubule release in the first instance. The least likely is that having released the polar body microtubules, both sets of kinetochores drop the egg pole attachment and cause 0:4 attachment. Other explanations for polar body biased events (0:4, 1:3) include a selfish centromere that erroneously attempts to flip when egg bound. Selection of all forms should minimize this error. The above model predicts early release of polar body microtubule attachments.

This logic of the selfish aneuploid-inducing centromere has precedence. It bears resemblance to the theory for the invasion of cytoplasmic male killers, seen in insects [55,56]. Here, cytoplasmic factors also act conditionally on being in a genetic dead end (the male) by killing and allowing resource distribution to those with their clonal relatives that are not a dead end (the same cytoplasmic factor in females) [55,56]. The death of males can, for example, reduce competition for resources or provide a first meal for cannibalistic ladybirds [57]. Related effects may be seen in flowering plants. Here, a diploid mother cell produces 4 products, only one of which is a functional (germinal) egg. Selection can then favour alleles expressed in somatic spores that interfere with the development of their sister germinal ovule if this benefits other ovules [58]. Both cases require conditional behaviour by alleles that then benefit clonal relatives via compensation.

## Bub1 dosage effects are consistent with the selfish centromeric aneuploidy model

After invasion of an aneuploid-inducing centromere, selection for suppressors of the aneuploidy induction is likely. The invasion could lead to fixation or internal equilibrium depending on the fitness of the centromeric aneuploid-inducing homozygotes (S1 File). If the selfish centromere induces aneuploidy at the same rate as it does in the heterozygote and suffers some further fitness costs then, if invasion is possible, an internal equilibrium will be found (S1 File). If there is an internal equilibrium, invasion of suppressor alleles is all but inevitable. Even if selfish alleles transit towards fixation, unlinked suppressor alleles can be favoured while the selfish allele remains polymorphic. Once at fixation, there can be selection that reduces costs (cf. [59–61]).

A strong candidate suppressor locus is *Bub1*. The Bub1 protein is enriched on centromeres with more mitotic centromere-associated kinesis (MCAK) [41], and the *Bub1* axis is a known modulator of selfish-driving centromeres [41]. Importantly, a heterozygous *Bub1* mutation (reducing dosage of Bub1) causes aneuploidy in oocytes (but not sperm) of mice [15]. Consistent with release from suppression of the sort of aneuploid-inducing centromere that is envisaged, half of the embryos are killed by aneuploidy. The consistency assumes centromeric heterozygosity, which is likely as the strain employed, B6C3F1, is a between-strain hybrid. More indirectly, but also consistent with such an effect, BUB1 levels reduce with age [62] (N.B. this is not the only maternal age effect mechanism [4,13]).

## Selfish aneuploidy and maternal age effects

The reduction of BUB1 with age is potentially significant as there is an increasing trisomy rate with age [4,22,63]. In humans, the rate of meiosis I aneuploidy shows an inflection to accelerating rates at approximately age 35 [9]. Before that, the increase with age is relatively shallow [9]. In outbred Swiss CD1 mice, there is an increasing rate with age, but with no apparent inflection in aneuploidy rates in eggs [64], although data is more limited (and what happens in the wild is unknown).

Evolutionarily speaking, these increasing rates could be broadly explained by classical senescence theory [16], i.e., more effective selection operating on the young [29,65]. However, why aneuploidy doesn’t show a male age effect [66] (but does as regards nonaneuploid chromosomal abnormalities [67]) is then not so transparent. Might there be something peculiar about selfish MMI centromeric aneuploidy that provides the basis for an additional (complementary), or more specific, model for age dependency? Weakening selection for suppressors of selfish centromeric aneuploidy with age, for similar reasons to there being weaker selection with age on all traits [29,65], is one possibility. The declining levels of BUB1 with age [62] are consistent, but not uniquely so. That the age effect is owing mostly to changes in centromeric cohesion [13] is similarly consistent, but not uniquely so.

That mice and humans may show different age-related trends is itself intriguing and warrants further investigation. In the current context, the mode of compensation we also expect to be different as humans tend to have singleton births and mice have multiple offspring per brood. In mice, we expect the compensation to be immediate via redistribution of resources to surviving progeny. In humans, we expect the mode to be saved resources and faster time to the next reproductive effort.

In humans, we then also expect the extent of reproductive compensation to vary with maternal age as birth weight of the baby varies with age, with a peak at about the same maternal age as the inflection point of increased aneuploidy rates, i.e., 35 to 36 [68]. Assuming higher birth weight (within normal bounds) usually implies higher fitness, the profile of birth weights with age is such that there could be stronger selection for death by selfish aneuploidy in older mothers. This is because delay to reproduction in older mothers (i.e., non-induction of aneuploidy) is associated with reduced embryonic investment in the next pregnancy (approximately 4 years hence). In young mothers, where investment is higher in the future, the balance shifts the other way (S2 File). A complex demographic model incorporating other age effects (e.g., earlier reproduction means an intrinsically faster reproduction rate, etc.) would be a valuable follow-on theoretical analysis (cf. [69]). Any such model would need to also factor in that the rate of double ovulation increases with age in humans, also peaking in maternal mid-30s [69], probably as a means to reduce the costs of age-related reductions in embryonic viability [69]. Double ovulation increases the chances that one of the two embryos will be viable so altering the mode of reproductive compensation, making it more like the murine one. More generally, models comparing different modes of reproductive compensation might be especially informative.

## Among vertebrates, high rates of aneuploidy have only ever been observed in species with high reproductive compensation

The selfish aneuploidy model unusually predicts that MMI aneuploidy should be common when reproductive compensation for early mortality is high. Data to test for a relationship between reproductive compensation and aneuploidy rates are imperfect (S3 File). Nonetheless, the differences between aneuploidy rate estimates from mammals and other vertebrates (S3 File) are so large and consistent that methodological issues are unlikely to provide a full explanation. Specifically, in zebrafish, the per chromosome per embryo rate is approximately 0 [70], as it is in *Xenopus* [71]. Neither are expected to have reproductive compensation. In chickens [72,73] and zebra finches [74], trisomy rates are about 0.04% per chromosome per embryo. Limited post hatch compensation is possible in these species. By contrast, in all mammalian data (except inbred mice), there is an aneuploidy rate of approximately 1% per chromosome per embryo, this being seen in cows [10,75], pigs [76], hybrid mice [77], interstrain mouse crosses [15], outbred mouse lines [64], as well as humans [4]. The net percentage of aneuploid early embryos in a young female outbred mammal is thus approximately N%, where N is the haploid chromosome number (S3 File).

Inbred mice are a mammalian exception with very low aneuploidy rates (<1% per embryo)[77]. Many models could explain why this might be so. The current model would see that selection would not favour a selfish aneuploid-inducing centromere as the centromeric relatedness asymmetry no longer applies. Put differently, in an inbred lineage, any aneuploid-inducing centromere requires heterozygosity to invade and inbreeding forces homozygosity. Such models also presume that there were segregating centromeres with different propensities to induce aneuploidy in the ancestral population, but that selection that occurs during the creation of inbred lines will favour those for which homozygotes are fertile.

It is, however, also possible that there never were segregating centromeric variants that differ in their propensity to aneuploidy (although mice lines differ in the extent of aneuploidy [78] and aneuploidy is associated with centromeric variants—see above). Rather, we could just suppose that genomic homozygotes are intrinsically better at error-free meiosis and that the low rates are nothing to do with centromeric homozygosity per se. Perhaps, crossing-over homology searching is more reliable and well-formed chiasmata stabilise meiosis [79]? As MMI forces homology searching between the unrelated maternal and paternal chromosomes, as opposed to segregating the identical centromeres at MMII, such a model could explain why MMI is the source of most aneuploids.

Possibly, then, the more interesting question is why we see aneuploidy in some between-strain crosses [15,64] but less so in within-strain crosses [77]. Similarly, centromeric drive appears in between-species crosses [41]. One model evokes that they were always there, and present as variants in the wild population, just hidden in homozygotes. Alternatively, more heterozygous meiosis (not just centromeric) may just be just intrinsically more error-prone [79]. Were this the case the appearance of distortion/aneuploidy in outbred crosses would not be evidence for distorter alleles segregating in the ancestral wild populations. The same possibility applies to any distorters seen in hybrid/interstrain crosses and not in intrastrain crosses. A problem with the general homozygozity/heterozygozity model is the low rates of aneuploidy in fish [70], as these are relatively polymorphic, indeed more so than humans (S3 File). However, fish female meiosis is not stalled at MMI, so we may need to evoke a homo/hetero model that applies only to mammals as the null model. In some instances, hybrid disturbance affects male meiosis more than female meiosis, thus not obviously explaining maternal aneuploidy bias [80].

An important discriminating prediction would be what happens in mice that are homozygous at the centromere but heterozygous elsewhere, versus those heterozygous at the centromere but homozygous elsewhere. The selfish centromere model predicts low aneuploidy if the centromere is homozygous. The general heterozygosity model makes the opposite prediction. More generally if centromeric heterozygosity is the key, then we predict an order heterozygous everywhere (between strain crosses) > = homozygous everywhere other than the centromere > homozygous at the centromere, heterozygous elsewhere > = uniformly homozygous.

In sum, high aneuploidy rates have, in vertebrates, only been observed in outbred species with reproductive compensation, all of which are at least consistent with the selfish aneuploidy model. Alternative theories for aneuploidy coupled to drive [16,21,22] fail to explain this. One might, however, suggest that the selfish centromere model is unnecessary as reproductive compensation could enable aneuploidy to be maintained at high levels owing to mutation-selection equilibrium [33]. It is similarly expected to enable classical genetic diseases that kill us early in life to reach somewhat higher frequencies [33] and decrease selection against lethal recessives in consanguineous populations [81]. Consideration of a mendelian aneuploid-inducing mutation, as expected, finds that compensation acts to raise the equilibrium level (S1 File) (cf. [82]). Quantitatively, however, this seems unlikely to explain the very high rates observed (S1 File), not least because the relative fold increase for compensated autosomal lethals is of the order of 1.22, much less than for alleles of weaker effect [83]. Indeed, almost perfect compensation in mammals is needed to account for the difference in rates between fish (the best approximation to the zero-compensation condition) and mammals (S1 File). This result comes with the caveat that female meiosis in fish and mammals may be different and underlying rates affected by mammalian stalling at MMI (although why then inbred mice do not also show high aneuploidy rates requires further assumptions). Unlike the selfish centromere model, mutation-selection equilibrium models do not predict antagonistic coevolution.

## Further predictions and evidence

While suggestive none of this evidence is definitive. Further cross-species analysis of aneuploidy rates in early embryos of species differing in their level of reproductive compensation, and with known levels of in/outbreeding, is warranted. Analysis of species that are not domesticated or laboratory maintained would be especially valuable. Pairwise comparison of the few mammals with moderate degrees of incest (naked male rats, black-tailed prairie dogs, meerkats, and banded mongoose [84]) with nonincestuous sister taxa could prove valuable. As reproductive compensation is expected in placental fish and reptiles (squamates [85]), higher rates of MMI aneuploidy, compared with their nonplacental sister species [85,86], is predicted. More generally, if vivipary entails higher potential for reproductive compensation, then analysis of the more than 150 independent evolutions of this trait [87] would be informative. Birds too might be useful as the degree of reproductive compensation may well vary considerably depending on levels of parental care, sex biases in such care, and opportunities to reproduce again, etc.

Differences between singleton-birth mammals and multi-progeny per brood species in mode of compensation provide further testing opportunities. Not only might the trends with aging differ (see above), but in addition, the degree of fitness compensation is likely to be weaker in the multi-progeny species (redirection of resources) than the singletons (save resources and rapidly reproduce again). In mice and pigs, the per chromosome rates are a little below 1% per chromosome but higher in cows and humans (S3 File). However, these data are extremely noisy (method uncontrolled, relative age uncontrolled, etc.) and so this small possible difference cannot be given credence. A repeatable finding of well-controlled differences in oocyte rates would however be supportive of their being some role for reproductive compensation in the evolution of aneuploidy rates. Analysis of primates with multiple offspring per brood (e.g., some strepsirrhines [88]) with their singleton producing related species would be instructive.

The model also predicts bouts of invasion and suppression. It is thus compatible with recent centromeric selection in humans [45,46] and with rapid coevolution of centromeres and their interactors [17–20,41,48–51]. In the current model, antagonistic coevolution between centromeric aneuploid inducers and their suppressors is expected. Indeed, as centromeric drive (sensu strictu) isn’t associated with excess embryo mortality (just non-mendelian transmission ratios), selection against aneuploidy is probably considerably stronger than selection against pure centromeric drive. If so, we expect signals of antagonistic coevolution in species with reproductive compensation (mammals) and not so much in those without (e.g., free-spawning nonplacental fish), despite the same potential for centromeric drive in all species with asymmetric female meiosis.

The model also predicts higher aneuploidy rates in some non-vertebrate species. For example, assuming some aneuploid embryos die early, insect species with cytoplasmic male killers are predicted to have reproductive compensation [55,57]. At first sight, this may help explain why *Drosophila* has a female meiosis I nondisjunction rate [16] that is comparable to ours [89]. Many species of flies also have cytoplasmic male killers suggesting that they have reproductive compensation [55]. However, it seems unlikely that compensation in flies is anything like as strong as in mammals. The high rates in fruit flies are thus enigmatic and are quantitatively problematic for the current model.

The model can also be analysed by within-species analysis. There are multiple reasons to suppose that centromeric aneuploid inducers and their suppressors should be polymorphic (cf. [16]). If suppression is itself costly, for example, we expect the selfish element not to be fully suppressed/eliminated. In quantitative genetical terms, the model thus would be consistent with heritability of aneuploidy rates, seen in flies [16], and genetic background effects, seen in mice [78]. That women with especially high rates of chromosomal errors in meiosis typically have single chromosome aneuploidy as the cause [9] is also consistent. The model also predicts both centromeric *cis* effects and *trans* effects, the later mediated via proteins that interact with the centromere (e.g., Bub1 [15]). Given current very high levels of female meiosis I nondisjunction, chromosome 16 [11,89] may be a candidate for a selfish aneuploid-inducing centromere yet to be fully suppressed. If so, we predict a centromeric haplotype that induces aneuploidy when being moved to the polar body. It would then have disproportionate representation in aneuploid progeny.

The model may also make predictions about between-chromosome rates of aneuploidy. If all else is equal, the model predicts easier invasion of aneuploidy inducers for those chromosomes monosomics or trisomics of which kill earlier (i.e., typically larger chromosomes), so enabling more reproductive compensation. In young females (<20 years), chromosomes 1 to 5 have an aneuploidy rate of the order of 0.1 per oocyte, while smaller ones (chromosomes 13 to 15 and 21 to 22) have a rate approximately 0.02 per oocyte [54]. However, such data are not necessarily as supportive as they might at first appear. In younger women, larger chromosomes have high rates of 4:0 segregation (class 2, Fig 2), while shorter ones have proportionally more 3:1 (class 3, Fig 2) mis-segregation [54] suggesting different modes of aneuploidy and hence possibly different mutational opportunities for centromeres. Chromosomes also differ in the mode of aneuploidy with age, and the rate of change with age [54]. In 20 to 32 year old women, for example, rates for the large and small chromosomes are more comparable (approximately 0.02 for the large, approximately 0.04 for the small)[54]. Thus, the net effects will be sensitive to assumptions about the reproductive contribution of females of different ages. More generally, the mechanisms of aneuploidy are different for smaller and larger chromosomes [90], possibly owing to different cohesin densities, thereby confusing tests. For a few chromosomes, aneuploidy is more commonly a consequence of meiosis II effects [53].

These data also indicate the need for good oocyte estimates, not early embryo estimates, as there is a selective filter between oocyte and early embryo (N.B. preimplantation mortality of fertilised or unfertilised eggs can all give rise to compensation). For example, while aneuploidy is common for the larger human chromosomes in oocytes [54,91], owing to early selection this is not reflected in early embryonic data [91]. Similarly, some methods (e.g., FISH) report exceptionally high rates (>70%) of nondisjunction in eggs [2,3], consistent with very early stage (oocyte to zygote) mortality. While then some data suggest that large acrocentric chromosomes predominate as early embryonic aneuploids (e.g., in pigs [92]), and others suggest the opposite (in humans and cows) [4,10], inferences from such data are hard to draw.

The previously identified high oocyte rates [2,3] have been dismissed as biologically implausible [4]. Conversely, I suggest that they may be entirely plausible in a species, like us, with strong reproductive compensation. More generally, high rates of aneuploidy and the failure of so many pregnancies may be a price we pay for looking after our young so well.

## Supporting information

S1 File The population genetics of a selfish aneuploid inducer. Frequency of a mendelian aneuploid locus under mutation-selection equilibrium.
(PDF)

S2 File Maternal age effects and reproductive compensation. (PDF)

S3 File Evidence of aneuploidy rates across vertebrates. (PDF)
