# Supplementary material for: Selfish centromeres and the wastefulness of human reproduction
Source: PLoS Biol. 2022 Jul 5;20(7):e3001671. doi: 10.1371/journal.pbio.3001671 (PMC9255743; doi:10.1371/journal.pbio.3001671)
Supplement: S1 File — Frequency of a mendelian aneuploid locus under mutation-selection equilibrium. (PDF) [file pbio.3001671.s001.pdf]

Supplement 1: Invasion and equilibrium conditions for aneuploid-inducing centromeres are very broad

Here I consider the population genetics of the form of centromere under discussion. Given that we are here considering a system whose equilibrium (if there is one) will be dependent on costs and actions of drivers and of suppressors, rather than following a game theoretical approach [1] I instead start by considering invasion conditions of centromeric drivers.

Before going into the new model, lets first consider a classical distorting selfish centromere. This is helpful to clarify how this selfish aneuploidy model is different from classical selfish centromeres. I assume the ancestral state is fixation of a (presently) non-driving centromere (segregation is Mendelian) with organismic level fitness 1. Assuming no inbreeding, the new drive allele (*D*) appears in a heterozygote (*Dd*), so invasion conditions are dependent on heterozygous effects and wild type homozygous effects alone (mutant homozygous effects are not important as at invasion such homozygotes are not present). Let us assume that half of the time our centromere is "lucky" and is taken to the egg pole. The other half of the time it is taken towards the polar body but can flip k proportion of the time so getting transmitted, k running from 0, no distortion, to k=1, the only allele being transmitted is the selfish allele. The transmission rate of the selfish centromere is then $0.5 + 0.5\,k$ in the heterozygote. The fitness of heterozygotes is $(1-t)$, i.e. they have a cost to bearing the selfish centromere. Homozygotes for the new allele have fitness a. With p the frequency of the driving centromere (and q the frequency of wild type) in the current generation and p' in the next, then assuming Hardy-Weinberg equilibrium for fertilizations:

$$p' = [ap^2 + 2(0.5+0.5k)(1-t)pq]/w \text{ where } w = ap^2 + 2(1-t)pq + q^2 \qquad (1)$$

where w is the weighted mean fitness in the population. Notice here that I assume that whenever the selfish centromere isn't inherited the wild type allele is, hence the term $(1-t)pq$ in the mean fitness (I discuss this assumption below). We can consider invasion as satisfying the condition dp'/dp at $p=0 > 1$. For invasion thus,

$$(1 + k)(1-t) > 1 \qquad (2)$$

holds. Note that under classical Mendelian inheritance k=0 and so for invasion $(1-t)>1$ is required (i.e. the allele is advantageous). Conversely, a fully driving chromosome (k=1) can invade even it

reduces fitness by up to 1/2. This classic result [2-4] underscores the vulnerability of meiosis I in females to even highly costly distorting elements [see also 5].

Now let us consider a centromere capable of such conditional flipping, but sometimes failing to cleanly flip. Inequality 2 describes a case if every meiosis is either successful for the drive (flipping) chromosome or successful for the non-driving chromosome, with the "loser" going to the polar body (i.e. no aneuploidy, just segregation distortion). A case without aneuploidy, where the flip is successful and the selfish centromere goes to the egg, the alternative to the polar body, we can consider as a "clean" flip. What happens if a clean flip isn't seen? There are at least two possible resolutions: at a rate *j*, the embryo is aneuploid (probably trisomic given the nature of flipping to move towards the egg pole) or the centromere driver ends up in the polar body, the wild type allele going to the egg pole (at rate 1-*j*). In the second case, there is again no aneuploidy. This accords with this decision chart (in red is the only circumstance in which the selfish allele has no potential for further transmission):

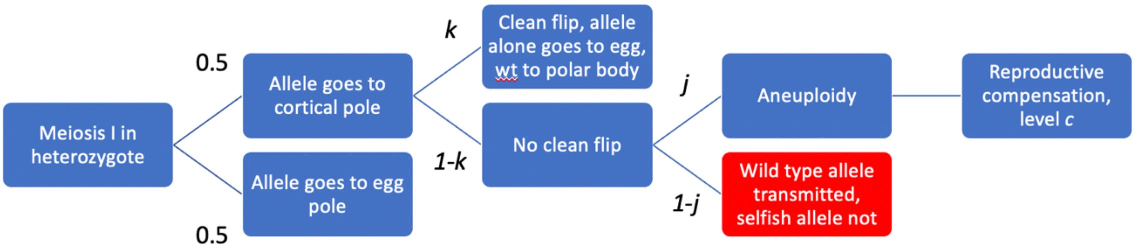

Importantly in mammals, when an aneuploid dies early, it frees the mother up to reproduce again and/or or redistribute the resources amongst the viable progeny i.e. reproductive compensation [6-8]. Let us suppose that death of the embryo allows compensation *c* (*c*, range zero, no compensation, to 1, perfect compensation). If we consider reallocation of resources within a brood (as in mice), then *c* would be dependent on the number of embryos dying from aneuploidy within the brood (i.e. $0.5(1-k)j$) and the number of embryos amongst which the released resources would relocated. Thus $c = \beta(1-k)j/(1+k + (1-k)(1-j))$, where β is some constant reflecting the efficiency of resource re-allocation, the effects this has on fitness. If embryo mortality occurs earlier rather than later, we expect this variable to be higher. If $\beta=0$ there is no reproductive compensation. Note that in this instance the benefits of embryonic mortality for the survivors accelerate with increasing mortality as more resources are released to be shared between fewer progeny.

A proportion *b* of that compensation goes to the selfish allele. If a mother reproduces again for example, then at least half the time the selfish allele will go the egg body. If so $b >= 0.5$. More

generally, we expect b to reflect the proportion of viable (non-aneuploid) offspring that have the selfish allele, this being $b = (1+k)/(1 + k + (1-k)(1-j))$. Rather than fully modelling these effects, in the first instance we may consider compensation's net effect on the selfish allele as $C = c.b$. If additionally, we again suppose there to be a cost, t, to bearing the drive chromosome as a heterozygote, then invasion requires:

$$2\,(1-t)\ \ (0.5 + 0.5.k + 0.5(1-k).j\,.C)) > 1. \qquad (3)$$

Note that this does not also factor in the effects of non-transmission of the wild type allele (in aneuploids) in the calculation of the mean population fitness. At invasion this is a good approximation as the net effect on the wild type allele through non-transmission is infinitesimally small when the mutant allele is infinitely rare [c.f. 9]. After the allele invades it will, however, have the secondary advantage of reducing the transmission rate of the wild type locus (see below).

For the condition that $j = 0$ or $c = 0$ (either no non-disjunction or no compensation) the condition resolves to the classical result above [2, 3] in which a degree of distortion that more than counterbalances the individual level selection i.e. $(1 + k)(1-t) > 1$, i.e. there must be some degree of segregation distortion.

However, what happens when aneuploidy is induced ($j > 0$)? Here invasion is possible even when there is no segregation distortion *sensu strictu*, $k = 0$. For the sake of clarity, let us suppose there is no immediate individual level cost to possession of the drive chromosome ($t=0$) to render the logic clearer. From inequality 3, even if the centromeric driver does not drive (half of the embryos have the mutant centromere alone) it can still invade so long as $j>0$, $C>0$. The reason for this is that the only embryos poisoned by the aneuploidy are those that would not have had the selfish driving centromere owing to the conditional nature of the flip. In humans, the poisoning by the aneuploidy (rate $0.5\,j$), forces earlier reproduction and redirection of resources that would have gone to the wild-type chromosome at rate C. In the subsequent reproduction the selfish centromere stands a further 50:50 chance of transmission. Such females in effect have more reproductive efforts and their viable reproductive efforts disproportionately transmit the selfish chromosome. In mice the more likely effect is redistribution of resources to viable brood mates, which disproportionately have the selfish allele as $b = 1/(2-j)$ is greater than 0.5 if $j > 0$ i.e. if aneuploidy killing is happening.

Given the large advantage that can happen when j and c are both high, it is perhaps then no surprise that this advantage can outweigh substantial direct costs of bearing the aneuploidy allele. For the case of no segregation distortion *sensu strictu* (k=0), the invasion inequality resolves to:

$$1-t > 1/\ (1 + j.C) \tag{4}$$

If we take C=1, j=1 as the limit (half the brood are aneuploid with perfect compensation all directed to the selfish allele bearers), then the direct cost of bearing the selfish aneuploid inducing chromosome can go up to a 1/2 reduction in fitness and the aneuploid-inducing chromosome can still invade.

While it might seem peculiar that aneuploidy could ever be favoured by selection, a simple numerical example makes the point. Imagine a mother will raise only two children. Through early meiosis I the selfish aneuploid inducing centromere is the "lucky" chromosome half the time so on average goes to one of the two offspring. Thus, if it does nothing its rate of transmission is 0.5. Imagine however that during the half of the time it would not be transmitted it kills the surviving embryo by making it an aneuploid. The mother rapidly reproduces again. This time the chromosome has a further 50% chance of being transmitted giving a net average occupancy of 1.5 of the two children (b=0.5). Its net degree of transmission (per offspring) is thus 1.5/2=0.75, this being greater than the 0.5 expected under Mendelian inheritance. It might also be the case that if it is unsuccessful in this the subsequent conceptus then it can kill again and again until the two surviving progeny are reared (C>0.5, j=1). It could then be transmitted to both of the surviving progeny, while never formally causing centromeric drive in meiosis (k=0 all the time).

For the case of compensation via redistribution of resources to surviving sibs, we estimate (see below), that death of half the offspring gives a ~10% increase in survivorship to the survivors. Using β =0.1, then if k=0 and j=1, the selfish aneuploidy can invade so long as t< 0.09. That is to say, so long as the cost of possessing the selfish chromosome in a heterozygote, independent of its impact on aneuploidy is less than 0.09, realistically, selfish aneuploidy can evolve. This is very permissive. As the efficiency of aneuploidy decreases (j->0), so the maximum value of t also decreases towards zero.

Equilibrium post invasion

To consider the post invasion equilibrium we need to expand the specification of the mean fitness in the population to additionally allow for the fact that heterozygotes for the aneuploid inducer also reduce the transmission rate of the wild type allele. Thus, now we write:

$p' = [ap^2 + 2(1-t)pq\,[0.5 + 0.5.k + 0.5(1-k).j\,.c.b]/w$
$q' = [q^2 + 2(1-t)pq[0.5\,(1-k)(1-j) + 0.5(1-k).j\,.c(1-b)]/w$

where
$$w = ap^2 + 2(1-t)pq\,[\,0.5 + 0.5.k + 0.5(1-k).j\,.c + 0.5\,(1-k)(1-j)] \;+ q^2 \qquad (5)$$

the final term in the heterozygous component reflects absence of a clean flip (rate 0.5(1-k)) that resolves as transmission of the wild type allele rate (1-j), as opposed to aneuploidy, and destruction of the selfish centromere in the polar body. We also allow for the reproductive compensation to benefit the transmission of the wild type allele at a rate $c.(1-b)$.

If $D=(1-t)(1 + k + (1-k).j.c.b)$ and $E= (1-t)\,[\,1 + k + (1-k).j\,.c + (1-k)(1-j)]$ then equilibria in this system are found at $p^*=0$, $p^*=1$ and $p^*=(D-1)/(E-1-a)$.

Consider now the case that occurs when k=0, so the only advantage of the selfish centromere comes via reproductive compensation owing to induction of aneuploidy. In this case the third solution resolves to:

$$p^* = [(1-t)(1+jcb) -1]/[(1-t)(2+j(c-1)) -1 -a] \qquad (6)$$

If we consider the case where j =1 then b=1 also holds (the selfish centromere always induces aneuploidy and hence all of the redirected resources go to the selfish allele). Then

$$p^* = [(1-t)(1+c) -1]/[(1-t)(1+c) -1 -a] \qquad (7)$$

As homozygous fitness, a, must be equal to or greater than zero, in this instance there can exist no internal equilibrium, as the numerator will always be greater than (a>0) or equal to (a=0) the denominator. Let us, instead consider the case of incomplete aneuploidy induction (j<1, hence b = 1/(2-j)). If we take the case of 80% aneuploidy induction when the selfish allele is going to the polar body (j=0.8), then the compensation benefits go to the selfish allele 83% of the time. If we suppose compensation to make up for 50% of fitness loss (c=0.5), then invasion is possible if t <

0.25. Considering the bounds from zero, no cost in the heterozygote, to this limit, the equilibrium solution, as a function of homozygote fitness, *a*, is illustrated in A:

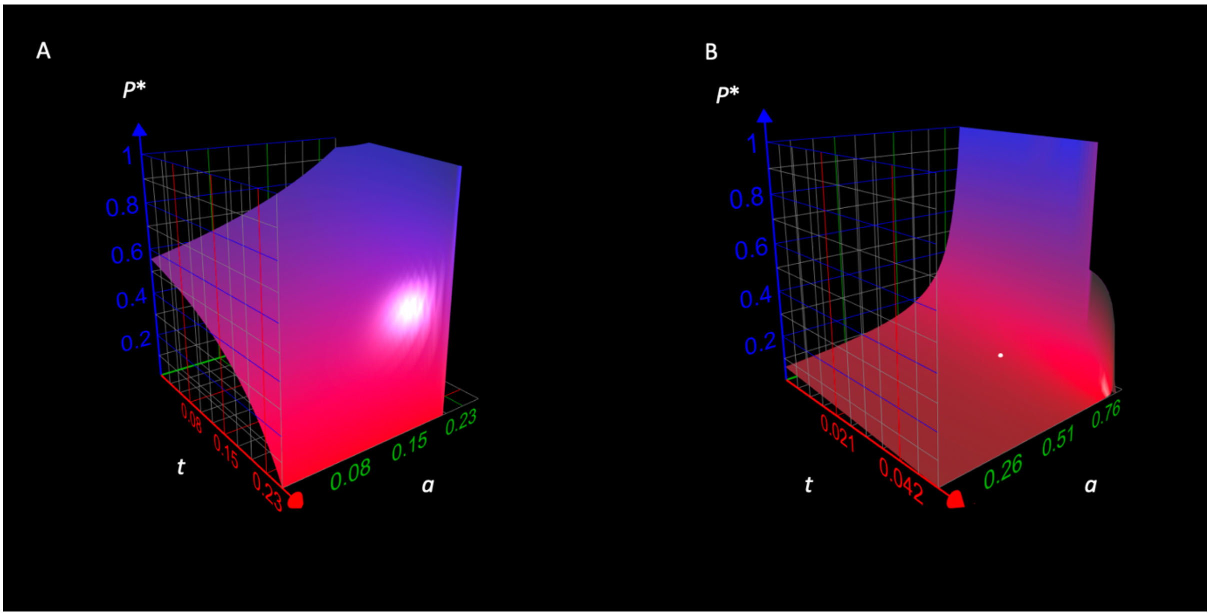

Invasion is followed by an internal equilibrium ($p^*<1$) when the selfish centromere homozygotes fitness is low ($a \to 0$). Low fitness could perhaps be because homozygotes are effectively infertile, always resulting in aneuploid offspring. A weaker aneuploid inducer (j=0.2, b=0.555), with the same compensation efficiency (c=0.5) can invade if t< 0.0527. The equilibria are shown in figure B, above. Here internal polymorphism is seen across a broader span of homozygous fitnesses. Note that for both figures invasion is possible throughout the space.

More generally, we can solve for the maximum value of homozygous fitness that is consistent with an internal equilibrium, p^* <1. Assuming $b = 1/(2-j)$, then this resolves as:

$$a < (1-t)(1+j(c - 1 - c/(2 - j))). \qquad (8)$$

We can then ask about the conditions for balanced polymorphism as a function of t and j, for various values of compensation, c. These are plotted below.

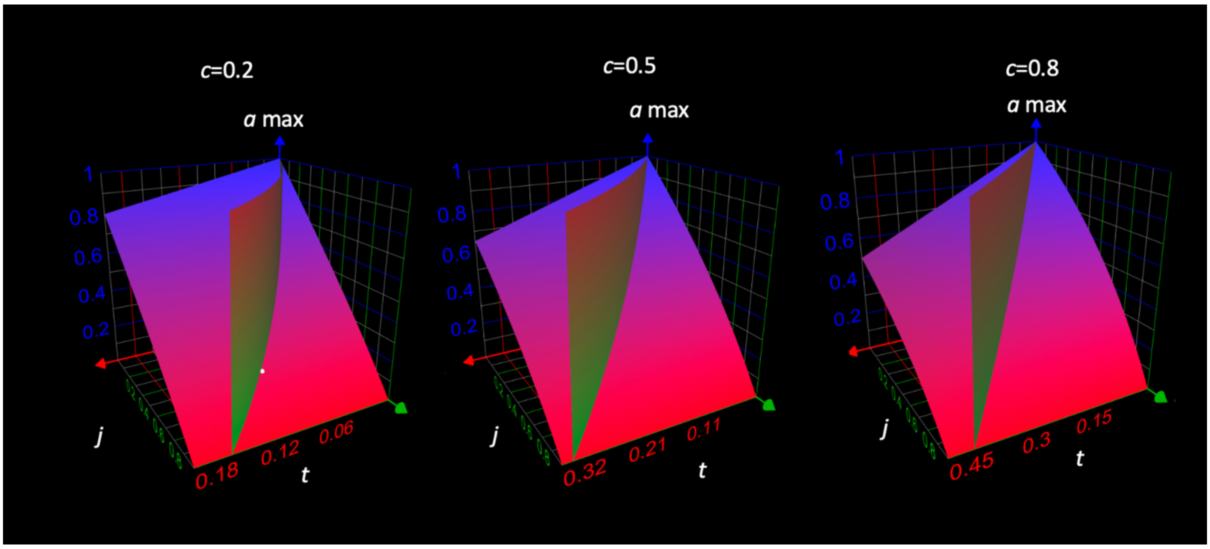

Here the positions to the right of the green divider are commensurate with invasion. Invasion space is more constrained as the benefit from aneuploidy induction decreases (*c*->0, note change in span of the *t* axis across different values of *c*) or the rate of aneuploidy induction declines (*j*->0) or the cost of bearing the allele (independent of aneuploidy costs) increases (*t* increasing). The maximum value of homozygous fitness (*a* max) commensurate with balanced polymorphism (red/blue sheet) for all incidences is greater than zero so long as $j < 1$. However, as the strength of aneuploidy induction increases (*j*->1), and the costs to bearing it reduce (*t*->0), there tends to be reduced possibilities for an internal equilibrium.

If we assume that the fitness of homozygotes is the probability of no aneuploid induction by either chromosome, i.e. 1-*j*, then at *a*=1-*j*, the condition for invasion is exactly the critical value for polymorphism to be seen. Solving for *t*, in the equation 1-*j*=(1-*t*) (1+ *j*(*c*-1-1(2-*j*)) gives *t*= *j.c.*(2-*j*+*jc*). Similarly solving for invasion from (1-*t*)=(1/ (1 + *j.c*/(2-*j*)) provides the same solution. Hence if *a*=1-*j*, then if invasion is possible polymorphism isn't. However, if the fitness of homozygotes is (1-*j*)(1-*s*), then if invasion is possible, the homozygote fitness is such that an internal equilibrium will be found so long as *s*>0.

The maintenance of polymorphism when homozygous costs are higher, closely resembles what is seen with segregation distorters, such as the *t*-complex in mice and SD in flies, in which an internal equilibrium is found after invasion if homozygotes fitness is low [4].

Centromeres are the most likely agents to induce aneuploidy as maternal meiosis I is guaranteed to segregate unrelated centromeres owing to their lack of crossing over and they also have greater

opportunity as they attach the spindles that drag one way or the other. The present model could, however, be extended to non-centromeric effects. Chromosome 18 trisomics tend to be a product of meiosis II non-disjunction [10]. Telomeric aneuploidy induction might explain this if the crossing-over pattern in meiosis I is such that telomeres of these chromosomes tend commonly to be unrelated in meiosis II. However, mechanistically how this might be achieved is not so clear as telomeres don't attach spindles.

Conditional versus unconditional strategies

Above I have presented the logic for a conditional strategy: if heading to the polar body, force aneuploidy. Why not employ an unconditional strategy – always go to the egg? You will receive either drive or aneuploidy. Don't you win both ways?

The most obvious strategy to always get into the egg is to not sit on the meiotic plate. While some B chromosomes do this, and so drive given egg-polar body size asymmetries, this is not a viable strategy for A chromosomes. The consequence would likely be that partner A chromosome would also not sit on the plate as they need a partner. In this case all embryos will be aneuploids. As such there would be reproductive compensation but the next reproductive event would also be dud. The strategy would not be consistent with invasion as you need the partner chromosome to go the polar body half the time. For this you need to "play" the meiotic plate game to make sure that some of the time one of you is taken to the polar body.

An alternative unconditional strategy is to always go the egg and make sure the competitor goes to the polar body. This would be a cost free classical centromeric drive and run rapidly to fixation. This strategy also appears preferable to conditional flipping as a means to drive. Nonetheless conditional flipping has evolved.

A Mendelian aneuploid inducing mutation at mutation-selection equilibrium

Above I considered a non-Mendelian allele. What however, if instead we assume that aneuploidy is a consequence of a Mendelian allele. We expect such an allele to be maintained under mutation – selection equilibrium. Here I quantitatively consider whether this is a viable model to explain why there is a ~1% per chromosome per embryo aneuploidy rate in mammals (see supplement 3). To not confuse this model and the above models I change nomenclature. Here the aneuploidy allele I presume to be the rare allele and give it frequency q. I start by considering a dominant allele,

then ask about a recessive allele. I show that compensation does indeed raise equilibrium frequencies but argue that this cannot account for the observed levels. I provide estimate of the degree of compensation from human twin data and offspring survival by weight curves.

1. A dominant allele

Let us start by considering the allele is dominant so the same proportion die in a homozygote. In a heterozygote we thus assume that a proportion n of progeny die owing to aneuploidy and 1-n survive. Half of the survivors inherit the allele (N.B. this is key difference between this model and the above model). If q is the frequency of this allele then:

$$q' = (q^2 (1-n) (1 + R.n/(1-n)) + pq (1-n) (1 + R.n/(1-n)))/\mathrm{w} + p\mu$$

where μ is the mutation rate from the wild type allele (frequency p) to the mutant and R is some function relating resources released to the increment in fitness of survivors. If n progeny die, $R.n$ is a function indicating resources (fitness) released, which is shared between 1-n surviving progeny. At the very limit, R=1, i.e. each death is perfectly compensated, the allele is neutral $((1-n)(1+n/(1-n)))$ =1. However, this reflects the fact that when, in the above formulation, for example, half the brood dies, the remaining half double in size, but there are half of them. Human twin data indicates that on death of one twin the increment in size is more like 40% not 100%: 3296 g -2336g /2336 = 0.41, data from [11]. More particularly, doubling in size almost certainly does not mean doubling in fitness (probably considerably lower). For the mean weights of singleton and twin babies, using data from Karn and Penrose [12] (from their Fig 3), we can approximate the difference in survivorship, albeit with the caveat this these are figures for relatively modern society. Babies around 3296g have a ~98% chance of surviving while those 2336 have a ~90% chance. Thus, the compensation in fitness terms is of the order of 0.08/0.9 = ~9%.

Although this does not allow for the alternative mode of compensation, namely more rapid reproduction and saved resources following death of a singleton, it seems reasonable to restrict consideration to cases where R<1, i.e. death of an individual is not fully compensated. In this instance, the allele is a standard dominant deleterious allele. In the more conventional nomenclature of a dominant deleterious allele the fitness of homozygotes and heterozygotes is 1-s. In the above, then:

$$(1-n)\ (1 + R.n/(1-n)) = 1-s,$$

And hence $s= n(1-R)$.

If R=0 there is not compensation and so the deleterious effects are simply the proportion of embryos killed. In the absence of near perfect compensation, the allele thus behaves as a standard deleterious Mendelian allele, in which case mutation selection equilibrium is at:

$q^* \cong 2\mu/n(1\text{-}R)$.

Thus, owing to reproductive compensation, the equilibrium frequency is raised. If, for example, death of an aneuploid allows half the fitness to be reallocated (R=0.5), then the equilibrium goes from $2\mu/n$ in the case of no compensation, to $4\mu/n$.

As the aneuploidy rate is mammals is ≅ 1% per chromosome per embryo (see Supplement 3), we can ask what sort of mutation rates of the locus are required to explain observations were the effect owing to mutation-selection equilibrium alone. The total aneuploidy rate at equilibrium is:

$n.q^{*2} + 2.n(1\text{-}q^*)q^*=0.01$

Assuming that p~1 and hence that most aneuploidy occurs in heterozygotes, then $4.\mu/(1\text{-}R) \cong 0.01$. One issue here is that, given the human mutation rate, the size of the locus controlling aneuploidy seems to have to be unusually large. Assuming a good degree of compensation (R=0.1-0.5), the relevant locus's mutation rate needs to be of the order of 10^{-3}. Given that the human per bp mutation rate is 2×10^{-8} ber bp, the locus concerned needs to be very large having 10^5 sites any of which, if mutated, induce aneuploidy. If R=0.9, this becomes ~10^4 sites. If we suppose there to be only a small proportion of sites in any gene to have such an effect, the gene size issue is yet more profound.

The above approach has a problem in that we must assume a source of mutations and cannot be sure about R. An alternative approach is to ask what R must be to account for the difference between mammals and fish. If aneuploidy were held at mutation-selection equilibrium, then in fish we expect to see aneuploids at some appreciable rate, if all else is equal (other than compensation). If R = 0.5 for mammals, for example, fish (R=0) should have a rate approximately half the human rate (i.e. around 0.005 per chromosome). Even if we assume that the very next embryo to be

observed in the fish study were to be aneuploid, we have a rate of 2.8 x 10^{-5} per chromosome per generation, over two orders of magnitude lower than the necessary figure. Even if compensation were very high, R=0.9, then we still expect to see fish aneuploids an order of magnitude rarer than in mammals (i.e. around 0.001), which is still an order of magnitude higher than observed. Perhaps best, we can also ask what value of R is necessary to account for the difference. If all of the difference between mammals and fish is owing to different levels of compensation in mammals, with R=0 in fish, near perfect compensation (R = 0.997) is required in mammals to explain the difference under a simple mutation selection model. This is biologically implausible. Moreover, as this assumes the next zygote to be observed in fish is aneuploid, even with the highest estimate for fish, the rates seem far below what is expected if the mammalian rate is high, solely owing to mutation-selection equilibrium. This later comparison has the further advantage that we need not make any assumptions about the causative mutations, just that they are similar in fish and mammals.

2. A recessive allele

If we assume the allele is recessive, then all aneuploidy occurs in homozygotes. If so:

$$q' = (q^2 (1-n) (1 + R.n/(1-n)) + pq))/\mathrm{w} + p\mu$$

Again, this equivalent to the classic model in which fitness of homozygotes is 1-s and s= n(1-R). Mutation selection equilibrium for the allele is at $q^* = (\mu/s)^{1/2}$ and the frequency of aneuploidy at equilibrium is $n.q^{*2} = n. (\mu/s) = \mu/(1-R)$. This is approximately one quarter the rate for a dominant and scales in the same manner with R, hence to a first approximation the quantitative problems outlined above apply in the case of a recessive allele. The number of bases pairs (L), mutation in which lead to aneuploidy, needed to explain a rate of 0.01 is for example $L=0.01(1-R)/(2.10^{-8})$. Even with strong compensation (R=0.9) this is of the order of 50,000 base pairs. If R =0.1 (see above), then $n.q^{*2}$=1.11. μ. Assuming the mutation rate to be the same in fish as in mammals, such an effect cannot explain the orders of magnitude difference in aneuploidy rates.

We conclude that reproductive compensation will tend to increase the mutation-selection equilibrium level of a Mendelian locus inducing aneuploidy, but is not obviously a viable process to explain the high observed per chromosome rates seen in mammals.

1. Day T, Taylor PD. Chromosomal drive and the evolution of meiotic nondisjunction and trisomy in humans. Proceedings Of the National Academy Of Sciences Of the United States Of America. 1998;95:2361-5.
2. Sandler L, Novitski E. Meiotic drive as an evolutionary force. Am Nat. 1957;41:105-10.
3. Prout T. Some effects of variations in the segregation ratio and of selection on the frequency of alleles under random mating. Acta Genet Stat Med. 1953;4(2-3):148-51. Epub 1953/01/01. PubMed PMID: 13137883.
4. Hartl D. Analysis of a general population genetic model of meiotic drive. Evolution. 1970:538-45.
5. Östergren G. Parasitic nature of extra fragment chromosomes. Botaniska Notiser. 1945;2:157-63.
6. Hastings IM. Reproductive compensation and human genetic disease. Genet Res. 2001;77(3):277-83. Epub 2001/08/07. doi: 10.1017/s0016672301004992. PubMed PMID: 11486510.
7. Hastings IM. Models of human genetic disease: how biased are the standard formulae? Genetics Research. 2000;75(1):107-14. doi: Doi 10.1017/S0016672399003961. PubMed PMID: WOS:000086118600011.
8. Hamilton W. The moulding of senescence by natural selection. J Theor Biol. 1966;12:12-45.
9. Butcher DL, Deng H-W. Hypothetical SisterKiller. Nature. 1994;369:26.
10. Nagaoka SI, Hassold TJ, Hunt PA. Human aneuploidy: mechanisms and new insights into an age-old problem. Nat Rev Genet. 2012;13(7):493-504. Epub 2012/06/19. doi: 10.1038/nrg3245. PubMed PMID: 22705668; PubMed Central PMCID: PMCPMC3551553.
11. Martin JA, Hamilton BE, Osterman MJK, Driscoll AK, Drake P. Births: Final Data for 2016. Natl Vital Stat Rep. 2018;67(1):1-55. Epub 2018/05/19. PubMed PMID: 29775434.
12. Karn MN, Penrose LS. Birth weight and gestation time in relation to maternal age, parity and infant survival. Ann Eugen. 1951;16(2):147-64. Epub 1951/09/01. PubMed PMID: 14885877.
