# Supplementary material for: Selfish centromeres and the wastefulness of human reproduction
Source: PLoS Biol. 2022 Jul 5;20(7):e3001671. doi: 10.1371/journal.pbio.3001671 (PMC9255743; doi:10.1371/journal.pbio.3001671)
Supplement: S2 File — (PDF) [file pbio.3001671.s002.pdf]

Supplement 2: A peak in human birth weight at 36 selects for accelerating selfish aneuploidy in the older mother

A peak in human birth weight at maternal age 36 [1] alters the degree of benefit from reproductive compensation. This could explain the form of the curve relating maternal age to aneuploidy rates, accelerating after age 35. Assuming higher birth weight usually implies higher fitness (within normal bounds), the profile of birth weights with age is such that there would be stronger selection for death by aneuploidy in older mothers. This is because delay to reproduction in older mothers is associated with reduced embryonic investment hence selecting for induction of immediate embryonic mortality. In young mothers where future investment is higher the balance shifts the other way.

To see why, consider this problem. At any point in time, a selfish centromere in maternal meiosis I has one of two options: 1) kill the embryo now by induction of aneuploidy, forcing an immediate reproductive attempt or 2) go to the polar body when attached (by chance) to the polar body's spindle, so permitting the current reproductive effort but with a possibility of future reproductive efforts. In both cases the centromere has a 50% chance of being in the next progeny. Thus, a key variable is the different investment in progeny now, versus sometime in the future. The form of the curve relating birth weight to maternal age will thus affect the evolutionary relative success of the two strategies. Importantly, when a mother is young, the investment into progeny approximately 4 years in the future (the human inter-birth interval being 43 months [2]) is higher than investment now and dangerously low birth weights in young mothers [1] are more likely to be avoided. This will reduce selection favouring the "kill now and enable an early new pregnancy" strategy and relatively favour strategy 2 (go to the polar body). Conversely, as females grow older, the investment into future progeny will be lower than would be available now, so the "kill now and enable an early new pregnancy" is selectively more favourable compared to what it was in young women.

At around 35 years the average weight of a baby is the same as that at around 35 +4 years [1]. This thus is the inflection point where selection should increasingly favour strategy 1, "kill now and enable an early new pregnancy". This is by no means the full necessary calculation as earlier reproduction should also be favoured by a higher intrinsic transmission rate per unit time (a Malthusian parameter) and by reduced probability of extrinsic mortality (the probability that a mother is alive today must be higher than the probability that the same mother is alive any time in

the future). Nonetheless, that the meiotic I aneuploidy rate in human females shows an inflection to higher rates also at around age 35 [3] may be explainable.

1. Wang S, Yang L, Shang L, Yang W, Qi C, Huang L, et al. Changing trends of birth weight with maternal age: a cross-sectional study in Xi'an city of Northwestern China. BMC Pregnancy Childbirth. 2020;20(1):744. doi: 10.1186/s12884-020-03445-2.
2. Galdikas BM, Wood JW. Birth spacing patterns in humans and apes. Am J Phys Anthropol. 1990;83(2):185-91. Epub 1990/10/01. doi: 10.1002/ajpa.1330830207. PubMed PMID: 2248378.
3. Tyc KM, McCoy RC, Schindler K, Xing J. Mathematical modeling of human oocyte aneuploidy. Proc Natl Acad Sci U S A. 2020;117(19):10455-64. Epub 2020/05/01. doi: 10.1073/pnas.1912853117. PubMed PMID: 32350135; PubMed Central PMCID: PMCPMC7229693.
