# Supplementary material for: Selfish centromeres and the wastefulness of human reproduction
Source: PLoS Biol. 2022 Jul 5;20(7):e3001671. doi: 10.1371/journal.pbio.3001671 (PMC9255743; doi:10.1371/journal.pbio.3001671)
Supplement: S3 File — (PDF) [file pbio.3001671.s003.pdf]

Supplement 3: Aneuploidy rates in vertebrates

Data on aneuploidy rates is imperfect. It is hard to distinguish early mitotic errors from meiotic ones, IVF (or related technologies) may affect zygotes [1] (but similar numbers of meiotic aneuploids are seen in *in vivo* and in *in vitro* analysis in cows [2]) and, at least in humans, analysis of IVF embryos may be subject to ascertainment bias. Furthermore, rate estimates may depend on maternal age [3] and, as aneuploidy tends to be embryonically lethal [4], contingent on how early the embryos are examined. Inbreeding of lab maintained stocks may also force homozygosity and a more harmonious meiosis which need not reflect rates in the wild. With these caveats I consider the available data.

Zebrafish lay eggs which require no parental care [5] and disperse after birth thus likely avoiding intra-brood competition. Analysis of fish strain EKW indicates that of 455 cases with informative polymorphisms there were no incidences of chromosome gain from the mother and of 935 instances no cases of chromosome loss from females [see Fig 4C in 6]. This is consistent with the prediction of negligible aneuploidy and an estimate of a per chromosome rate of approximately zero. The strain employed is the most diverse of all zebrafish lab strains and as diverse as some wild caught populations [7] that are typically assumed to be outbred [8]. The diversity in EKW is higher than that of humans [8]. Inbreeding and associated homozygosity is thus unlikely to explain the low rates.

In egg laying *Xenopus* (N=18) chromosome nondisjunction leading to gain of a chromosome in oocyte meiosis I is "very rare", with none observed in 204 instances, giving an upper 95% confidence interval of less than 2% [9]. Again, the per chromosome rate is thus estimated to be around zero. In this instance the source stock was supplied directly from a company and there was no indication of heterozygosity or breeding program. Thus we cannot be sure that this low rate is not owing to homozygosity.

In birds there also is little potential for early reproductive compensation as embryonic provisioning is fixed (yolk allocation) and birds will sit on inviable eggs. In the well-studied species for which trisomy data is available (chickens and zebra finches), the mother lays a clutch of eggs that will all be incubated no matter whether some are inviable [10]. In comparison to comparable multi-offspring per brood females (mice, pigs etc), these birds save neither resources (the resources in the egg are given in advance) nor time (the clutch will need incubating). Energetic costs of egg production and incubation are considerable in both chickens and zebra finches, especially at lower

temperatures (see [10] and refs therein). Parental care might however enable some degree of compensation. Overall expectations are again that rates should be below human rates. Of 4182 four day chick embryos examined only 0.2% were trisomics [11]. With four chromosomes examined this provides an estimate of 0.05% per chromosome. Similarly, with 9 chromosomes analysed and a trisomic rate of 0.33% of embryos, Fechheimer's analysis (of slightly earlier embryos) suggest trisomy at about 0.04% per chromosome per embryo [12]. The 2N-1 rate is a third lower. Not all of these are maternally derived. The level of inbreeding is unclear and analysis was done visually.

These numbers are in accord with a microsatellite-based assessment of 6 of 40 chromosomes in zebra finches with 857 embryos culled after 4 days of artificial incubation. Here 0.6% had one or more loci with three alleles [13]. Of affected individuals, 44% showed evidence for trisomy, thus we estimate a rate of 0.04% per large chromosome, not all of which were from female meiosis (6 of 8, hence ~0.03% trisomy per chromosome of maternal origin). In the case of zebra finches the trisomy identification was done genetically with stocks maintained to avoid inbreeding as this is known to cause high embryonic mortality rates [13].

None of the bird estimate provides data on embryo level trisomy as most bird chromosomes are tiny. The zebra finch work was extrapolated to estimate as an upper bound [13]. Trisomics explain no more than a quarter of embryonic deaths (this is quite liberal as many chromosomes are very small and possibly incompatible with embryonic mortality in trisomy) [13]. With approximately 17% early embryonic mortality in this species in the wild, this suggests perhaps a trisomy rate no higher than 4% (some proportion of which are female meiosis I aneuploids). Thus, despite their very high haploid numbers in birds embryonic aneuploidy rates must be substantially lower than the human rates (20—40%) on both a per embryo and, most especially, on per chromosome level (0.04% in birds, ~1-2% in humans).

By the reproductive compensation argument, in contrast to the above cases where we see low levels of aneuploidy, cows should be closer to the human range with both comparable pregnancy, parental care and single offspring per brood. Cows do indeed have high aneuploidy rates, also in the 25-40% range, nearly all derived from meiosis I trisomy in females [14, 15]. This comes with the caveat that these are in vitro fertilizations (but see [2]). As required by the model, trisomics are much less likely to be brought to term [14] thus enabling reproductive compensation. These rates

are thus comparable to humans on both a per chromosome (N=30, rate ~0.8-1.3%) and per embryo rate.

In cross bred pigs rates may be slightly lower (11 of 77 =14.3% of early embryos are aneuploids [16], 10.4% of which involved loss/gain of one or a few chromosomes as opposed to more extensive imbalance). This also declines rapidly in slightly older embryos indicative of inviability [17]. With a haploid chromosome number of just N=18, the slightly lower rate may reflect approximate equality across mammals for the per chromosome rate (~1%). For porcine chromosomes 1 and 10 there is a 1.8% trisomy rate [18], consistent with a ~1% per chromosome rate.

In principle data from mice could be revealing. Laboratory mice are highly inbred while the above models have assumed an absence of inbreeding. At extreme inbreeding levels a selfish centromere is likely to be partnered with a clonal relative not an unrelated version. This being so, selection to poison the embryo should be nullified. More mechanistically, with no differences between the maternal and paternal centromeres preferential attachment to a spindle in female meiosis I seems difficult. The model thus predicts lower aneuploidy rates in inbred species. Consistent with expectations, in inbred mice aneuploidy rates are very low (<1%)[19]. By contrast, from hybrid mice (the best model we have for intra-specific outbred mice) rates are comparable to humans (~20%) [19] especially when viewed on the per chromosome basis (N=20, rate ~1% per chromosome). In the oocytes of old female mice aneuploidy rates are as high as 60% [20], also comparable with humans. In between-strain intra-specific crosses aneuploidy rates of the order of 15% are reported (WT female crosses in Table 2 in [21]), most of which are MMI derived [21].

Thus, available data suggests that in outbred mammalian early embryos there is an approximately constant mean rate of ~1% aneuploidy per chromosome, giving an embryonic aneuploidy rate ~N% where N is the haploid number. While every estimate can be questioned given the heterogeneity of experimental set ups, the rates are more than an order of magnitude higher than in other vertebrates. Broadly speaking the high aneuploidy rate in humans, and outbred mammals more generally, may thus be coupled to our ability to provide reproductive compensation owing to long pregnancy and our highly prolonged period of post-natal weaning.

1. Coppola G, Alexander B, Di Berardino D, St John E, Basrur PK, King WA. Use of cross-species in-situ hybridization (ZOO-FISH) to assess chromosome abnormalities in day-6 in-

vivo- or in-vitro-produced sheep embryos. Chromosome Research. 2007;15(3):399-408. doi: 10.1007/s10577-007-1125-2. PubMed PMID: WOS:000246222300011.
2. Tšuiko O, Catteeuw M, Zamani Esteki M, Destouni A, Bogado Pascottini O, Besenfelder U, et al. Genome stability of bovine in vivo-conceived cleavage-stage embryos is higher compared to in vitro-produced embryos. Human Reproduction. 2017;32(11):2348-57. doi: 10.1093/humrep/dex286.
3. Tyc KM, McCoy RC, Schindler K, Xing J. Mathematical modeling of human oocyte aneuploidy. Proc Natl Acad Sci U S A. 2020;117(19):10455-64. Epub 2020/05/01. doi: 10.1073/pnas.1912853117. PubMed PMID: 32350135; PubMed Central PMCID: PMCPMC7229693.
4. Nagaoka SI, Hassold TJ, Hunt PA. Human aneuploidy: mechanisms and new insights into an age-old problem. Nat Rev Genet. 2012;13(7):493-504. Epub 2012/06/19. doi: 10.1038/nrg3245. PubMed PMID: 22705668; PubMed Central PMCID: PMCPMC3551553.
5. Spence R, Gerlach G, Lawrence C, Smith C. The behaviour and ecology of the zebrafish, Danio rerio. Biol Rev Camb Philos Soc. 2008;83(1):13-34. Epub 2007/12/21. doi: 10.1111/j.1469-185X.2007.00030.x. PubMed PMID: 18093234.
6. Poss KD, Nechiporuk A, Stringer KF, Lee C, Keating MT. Germ cell aneuploidy in zebrafish with mutations in the mitotic checkpoint gene mps1. Genes Dev. 2004;18(13):1527-32. Epub 2004/07/03. doi: 10.1101/gad.1182604. PubMed PMID: 15231734; PubMed Central PMCID: PMCPMC443515.
7. Suurväli J, Whiteley AR, Zheng Y, Gharbi K, Leptin M, Wiehe T. The Laboratory Domestication of Zebrafish: From Diverse Populations to Inbred Substrains. Molecular Biology and Evolution. 2020;37(4):1056-69. doi: 10.1093/molbev/msz289.
8. Leffler EM, Bullaughey K, Matute DR, Meyer WK, Segurel L, Venkat A, et al. Revisiting an old riddle: what determines genetic diversity levels within species? PLoS Biol. 2012;10(9):e1001388. Epub 2012/09/18. doi: 10.1371/journal.pbio.1001388. PubMed PMID: 22984349; PubMed Central PMCID: PMCPMC3439417.
9. Liu D, Shao H, Wang H, Liu XJ. Meiosis I in Xenopus oocytes is not error-prone despite lacking spindle assembly checkpoint. Cell Cycle. 2014;13(10):1602-6. Epub 2014/03/22. doi: 10.4161/cc.28562. PubMed PMID: 24646611; PubMed Central PMCID: PMCPMC4050165.
10. El-Wailly AJ. Energy Requirements for Egg-Laying and Incubation in the Zebra Finch, Poephila Guttata. Condor. 1965;68:582-94.
11. Bloom SE. Chromosome abnormalities in chicken (Gallus domesticus) embryos: Types, frequencies and phenotypic effects. Chromosoma. 1972;37(3):309-26. doi: 10.1007/BF00319873.
12. Fechheimer NS. Origins of Heteroploidy in Chicken Embryos. Poult Sci. 1981;60(7):1365-71. doi: DOI 10.3382/ps.0601365. PubMed PMID: WOS:A1981LY78700004.
13. Forstmeier W, Ellegren H. Trisomy and triploidy are sources of embryo mortality in the zebra finch. Proc Biol Sci. 2010;277(1694):2655-60. Epub 2010/05/07. doi: 10.1098/rspb.2010.0394. PubMed PMID: 20444723; PubMed Central PMCID: PMCPMC2982043.
14. Silvestri G, Canedo-Ribeiro C, Serrano-Albal M, Labrecque R, Blondin P, Larmer SG, et al. Preimplantation Genetic Testing for Aneuploidy Improves Live Birth Rates with In Vitro Produced Bovine Embryos: A Blind Retrospective Study. Cells. 2021;10(9). Epub 2021/09/29. doi: 10.3390/cells10092284. PubMed PMID: 34571932; PubMed Central PMCID: PMCPMC8465548.

15. Nicodemo D, Pauciullo A, Cosenza G, Peretti V, Perucatti A, Di Meo GP, et al. Frequency of aneuploidy in in vitro-matured MII oocytes and corresponding first polar bodies in two dairy cattle (Bos taurus) breeds as determined by dual-color fluorescent in situ hybridization. Theriogenology. 2010;73(4):523-9. Epub 2009/12/22. doi: 10.1016/j.theriogenology.2009.10.007. PubMed PMID: 20022097.
16. Hornak M, Hulinska P, Musilova P, Kubickova S, Rubes J. Investigation of Chromosome Aneuploidies in Early Porcine Embryos Using Comparative Genomic Hybridization. Cytogenetic and Genome Research. 2009;126(1-2):210-6. doi: 10.1159/000245922. PubMed PMID: WOS:000272605100022.
17. Hornak M, Oracova E, Hulinska P, Urbankova L, Rubes J. Aneuploidy Detection in Pigs Using Comparative Genomic Hybridization: From the Oocytes to Blastocysts. PLoS One. 2012;7(1). doi: ARTN e30335
10.1371/journal.pone.0030335. PubMed PMID: WOS:000301570600032.
18. Zudova D, Rezacova O, Kubickova S, Rubes J. Aneuploidy detection in porcine embryos using fluorescence in situ hybridization. Cytogenetic and Genome Research. 2003;102(1-4):179-83. doi: 10.1159/000075745. PubMed PMID: WOS:000220538500030.
19. Koehler KE, Schrump SE, Cherry JP, Hassold TJ, Hunt PA. Near-human aneuploidy levels in female mice with homeologous chromosomes. Curr Biol. 2006;16(15):R579-80. Epub 2006/08/08. doi: 10.1016/j.cub.2006.07.018. PubMed PMID: 16890511.
20. Jones KT, Lane SIR. Molecular causes of aneuploidy in mammalian eggs. Development. 2013;140(18):3719-30. doi: 10.1242/dev.090589.
21. Leland S, Nagarajan P, Polyzos A, Thomas S, Samaan G, Donnell R, et al. Heterozygosity for a Bub1 mutation causes female-specific germ cell aneuploidy in mice. Proceedings of the National Academy of Sciences. 2009;106(31):12776-81. doi: 10.1073/pnas.0903075106.
